# Antenatal care as a risk factor for caesarean section: a case study in Brazil

**DOI:** 10.1186/s12884-022-05008-z

**Published:** 2022-09-25

**Authors:** Márcia Regina Cangiani Fabbro, Monika Wernet, Nayara Girardi Baraldi, Jamile Claro de Castro Bussadori, Natália Rejane Salim, Bernardino Geraldo Alves Souto, Andréa dos Reis Fermiano

**Affiliations:** 1 Federal University of São Carlos, São Carlos, Brazil; 2 University of São Paulo, São Paulo, Brazil

**Keywords:** Antenatal care, Pregnancy, Nurses, Nurse midwives, Primary health care, Caesarean section, Case studies, Quality health care

## Abstract

**Background:**

Antenatal care is an important tool to prevent complications and decrease the incidence of maternal and antenatal morbidity and mortality. In Brazil, quality, access, and coverage of antenatal care are described as insufficient. Consequently, high rates of caesarean section, congenital morbidities such as syphilis, maternal and early neonatal mortality occur, as well as obstetric violence and dissatisfaction with healthcare. It is important to reflect on health disparities in antenatal care. This study aimed to carry out a critical analysis of antenatal care in one city of São Paulo state in Brazil.

**Methods:**

A case study was performed, structured in a descriptive cross-sectional epidemiological study and two qualitative studies. Data for the epidemiological study was obtained from the Informatics Department of the Unified Health System (DATASUS) of Brazil, which was processed in the Epi-info v software 7.2. and treated descriptively and by the Mantel–Haenszel or Fisher's exact tests. Qualitative data was collected through semi-structured interviews with 30 pregnant women and 8 nurses in the primary healthcare service of one city in São Paulo. The qualitative data analysis was based on thematic content analysis.

**Results:**

The data revealed a limited quality of antenatal care. More than six antenatal visits increased the probability of a caesarean section by 47% and babies born vaginally had a lower Apgar score. There was little participation of nurses in antenatal care and women described it as “a quick medical appointment”, limited by protocols, based on procedures and insufficient in dialogue. Antenatal care appeared to be fragmented and permeated by challenges that involve the need for change in management, performance, and ongoing training of professionals, as well as in the guarantee of women’s rights.

**Conclusions:**

Caesarean section was statistically related to the number of antenatal care visits. Interactions between professionals and pregnant women were poor and resulted in dissatisfaction. There is an urgent need to connect health indicators with the findings from professionals and women’s experiences to improve the quality of antenatal care.

## Background

Despite health indicators showing improvement in perinatal care, it continues as a priority due to the unacceptable numbers of maternal and neonatal morbidity and mortality [1]. Thousands of women regardless of socioeconomic status, have their rights violated and experience mistreatment during pregnancy, childbirth and postpartum [2, 3].

High quality antenatal care is considered one of the most important and widespread tools in healthcare to promote positive pregnancy experiences and prevent maternal and neonatal morbidity and mortality worldwide. Antenatal care (ANC) has the potential to positively impact women’s health during pregnancy, birth, postpartum, and thereafter. Other aspects of women’s lives such as the economic, social, and cultural, must be considered and integrated to the biomedical perspectives to promote women’s health and wellbeing and have a positive impact on their self-esteem and autonomy [4].

The Brazilian Ministry of Health recommends that the first antenatal visit should occur before the 4th month of pregnancy, followed by six more visits before birth [5]. The Ministry commits to providing a humanized, safe, and high-quality care [6]. Yet, this does not always occur, and as a result, there are minimal changes to the maternal and neonatal mortality [7, 8].

The country is making efforts to improve the quality of antenatal care, mainly high-quality care standards, such as the Stork Network, “Rede Cegonha”, published in 2011 [9]. According to this document, primary healthcare is the gateway to the healthare system [9] and should provide access to other healthcare services, besides being responsible for the organisation and management of ANC. Despite the improvement in coverage, antenatal care is still described as inadequate and unequal [10], reflecting high rates of caesarean sections, congenital syphilis, maternal mortality, and early neonatal mortality [1, 11].

Although there is a strong global recommendation for nurses and midwives to participate in and provide ANC [4], “Birth in Brazil” a national survey with 23,940 women interviewed, showed that 75.6% of pregnant women were seen by a doctor [12], suggesting that there is an obstruction to midwives caring for women in the antenatal period.

Brazilian antenatal care needs to be reviewed to employ a holistic and humanistic approach (biopsychosocial medicine) as a counterpart to the traditional technocratic (biomedical) obstetric practice currently established. [13].

The connections within the healthcare system and the duties and responsibilities of each service involved in maternity care is another point that needs improvement [14, 15] to achieve a satisfactory pregnancy experience based on respectful, individualised, woman-centred care and supported by practitioners with good clinical and interpersonal skills [4].

This study analysed the ANC in a city in the state of São Paulo, Brazil, where the estimated population in 2018 was 249,415, and in 2021 was 256,915 inhabitants [16, 17]. The most recent data available on pregnancy-related deaths is from 2019, when the maternal mortality ratio was 67.54 per 100,000 live births. In 2018 when this study was conducted, it was 61,60 per 100,000 live births [18].

This study aimed to carry out a critical analysis of antenatal care in one city in the state of São Paulo, Brazil. Therefore, the following research questions were explored: "What are the indicators proposed by the Ministry of Health's programs and initiatives to evaluate antenatal and birth care in the city in question [5, 9] in 2018? How is care and the determinants of its quality perceived by health professionals and pregnant women?".

## Methods and materials

### Study design

To explore the case (antenatal care in one city of the state of São Paulo, Brazil), a cross-sectional, population-based, observational epidemiological study was carried out [19] integrated to two descriptive, exploratory, qualitative studies, one with pregnant women and another with nurses, from 2018 and 2019.

The case study method [20, 21] aims to direct the inductive processes of weaving the critical argument. It is an empirical investigation that addresses "how" or "why" questions concerning the phenomenon of interest by focusing on a singular situation, producing a rich and solid description of this phenomenon, and illuminating the reader's understanding of it.

### Study setting

The data was collected in a city with an estimated population of 251.983 people and an infant mortality rate of 6.42 deaths per thousand live births in 2019 [22].

In this city, the public health system is divided into five Regional Health Administrations. The ANC is developed in the primary healthcare, carried out in Basic Health Units (BHU). The ANC team consists of doctors, nurses, and nursing assistants, and the doctors are the main care providers to the pregnant women. There are not nurse-midwives in primary healthcare.

The Family Health Units (FHU) are composed of fixed teams: a general practitioner, a nurse, nursing assistants, and community health agents, who assist a specific region. Antenatal care is provided by the nurse and the doctor in interspersed appointments. This city had, at the time, 17 FHU and 12 BHU, and a Special Care Pregnancy Outpatient Clinic.

### Data collection and epidemiological analysis

In the epidemiological study, data from two sources were correlated: from the Live Births Information System (named SINASC by the Brazilian Ministry of Health); and the Mortality Information System (named SIM by the Brazilian Ministry of Health). SINASC contains information about access to antenatal care, births, and the immediate health conditions of newborns, provided by maternity hospitals at the time of birth. SIM receives data from death certificates completed by professionals who verify the information surrounding the deaths and contains sociodemographic data as well as causes of death. Consolidated national data from SIM and SINASC are publicly available for tabulation in the entry “Vital statistics” on the website of the Department of Informatics of the Unified Health System in Brazil—DATASUS [23–25].

The socio-demographic variables of women (marital status, age, education, and race/ethnicity), gestational variables (access to antenatal care, gestational age, number of antenatal visits and twin pregnancy) and birth conditions (type of birth, Apgar score in the first minute and newborn weight) were analysed.

A simple tabular description of all variables was made, followed by cross-tabulation of all variables with the type of delivery using 2 × 2 contingency tables. For statistical correlation inferences, the Mantel–Haenszel chi-square test was used, when the number of occurrences was greater than or equal to five, or Fisher's exact test when the number of occurrences was less than five. A value of *p* < 0.05 was assumed as an indicator of significance for the correlation analyses. Infant and maternal mortality indicators were also calculated.

### Data collection, Theoretical framework, and Qualitative analysis

The qualitative studies followed the Consolidated Criteria for Searching Qualitative Reports (COREQ). The theoretical framework was based on the conceptual basis of comprehensive care and on the concept of humanization of obstetric care present in policies and programs aimed at women's health in Brazil [1, 26–30]. The choice is due to the expanded conception of health and its scope in relation to the critique of the biomedical and technical model. It emphasises knowing and doing, that is, the "what" and "how" need to be considered to respond to the needs of the caregiver. It emphasises that the encounter in healthcare needs to be valued and requires a unique look, genuine dialogue, and inter-subjective exchanges, to preserve the meaning and scope of healthcare, which implies accepting the subjectivities of the actors involved, considering the technical and inter-subjective dimensions as structural elements of healthcare.

Semi-structured interviews were conducted with pregnant women (ANC in Brazil is mainly undertaken in BHU) and primary healthcare nursing professionals. Eligibility criteria were: 1) being a pregnant woman, over 18 years old, being cared for in a BHU and with a minimum of three antenatal visits at the time of data collection OR 2) being a nurse of a BHU in the city selected for the study. Exclusion criteria were: 1) being a pregnant woman with cognitive or linguistic difficulties that compromised the possibility of an interview.

The city had 12 BHU and 23 nurses were employed at the time of data collection. The municipal manager indicated six of them under the justification of being the ones with the highest number of antenatal care services. A research team member contacted the nurses of these units, explained the study, and verified their interest in participating. After confirmation, a date and time were scheduled for the interview, which took place in the unit, in a reserved room.

Women were invited to participate in the waiting room of the same six BHU. The woman who wanted to participate were invited to a closed room at the health unit where the study was explained again and her desire to participate in the study was verified. Once the participant accepted, the interview began. There were no refusals among the pregnant women invited to participate.

All interviews were one-on-one, semi-structured, audio-recorded, and conducted by a female researcher who was part of the research team. No members of the research team had relationships with participants prior to the study. The questions that guided the interview with the pregnant women were: "How do you perceive antenatal care?" and "What do you think could be improved and why?” For the professionals: "How do you perceive antenatal care in your unit?" "How have the nursing professionals been working in antenatal care?” The average duration of the interviews with the pregnant women was 25 minutes, and with the professionals, 30 minutes. For the sake of anonymity, the pregnant women were identified by the letter G followed by an Arabic number indicating their inclusion in the study.

The Thematic Content Analysis [31] directed the analytical processes of the empirical material resulting from the interviews. Initially, repeated readings of the transcribed material were made to grasp ideas, concepts, and themes. Next, new readings were conducted to highlight/select significant excerpts in terms of content in relation to the phenomenon being explored, which, when grouped together, indicated initial categories. Subsequently, interpretative movements and inferences articulated the data and delimited thematic categories to report the findings. No software supported the analysis.

Regarding the number of participants, by the 20th interview with the pregnant women, the data already pointed to saturation by meaning, that is, the set of data ensured enough elements in density and recurrence about the phenomenon under exploration [32]. However, the team decided to keep doing them in search for new elements, depth, scope, and diversity in the process of understanding pregnant women's perceptions about antenatal care. By the 30th interview these aspects had been contemplated, and the collection of qualitative data was concluded.

The number of professionals was based on convenience and initially, nurses from each unit were invited to participate in the study. If they declined, the manager nurses were invited since they also participated in antenatal care. All (nine) nurses of the units were invited, and eight of them agreed to participate in the study (some units had two nurses) and one declined the invitation.

All certified nurses in Brazil are legally allowed to provide antenatal care for low-risk pregnancies, and they can also take a postgraduate course to become nurse-midwives if they choose to become specialists. As of 2005, a direct-entry midwifery course was created. These midwives work in maternity hospitals, birth centres, maternity wards of general hospitals, and other sexual and reproductive health services.

Within the Brazilian primary health system there are different models of care. In the traditional model (Basic Health Units), doctors and nurses are not specialists in family health and the care is centralised in the triad: general physicians, obstetricians and paediatricians, and the professional providing ANC is the obstetrician. In the Family and Community Medicine model, nurses and doctors must complete a family health postgraduate course to work in the Family Health Program. In this model, antenatal care is provided by both nurses and physicians. Most nurses interviewed in this study are specialised in Family Health.

### Ethical considerations

All ethical recommendations were followed and respected, including the procedures related to the Informed Consent Form. This study had the approval of the ethics committee and is registered under the Certificate of Presentation for Ethical Appreciation (CAAE) number: 83424818.0.0000.5504, which also includes the approval for the interview with pregnant women, both carried out in 2018/2019.

## Results

The qualitative study brought the perspective of health professionals. The eight nurses interviewed were between 40 and 50 years old, most were female, all had more than ten years of professional experience in primary healthcare, two had specialisations in Women's Health and six were specialists in Public Health and Family Health.

The epidemiological study found that in 2018, 3,247 live infants were born to mothers residing in the city, almost all in a hospital setting (99.48%). The remaining were born at home (0.49%) or in another location (0.03%). These women were mostly Caucasian (68.09%), with secondary (high school) or tertiary education (college/university onwards) (58.88% and 27.84%, respectively), living in a stable marital union (79.09%), in the 20 to 34 age bracket (69.88%). From the gestational point of view, in most cases, the foetus was a singleton (96.61%) and was born at term (89.62%).

All participants had antenatal care that commenced in the first trimester and 75.27% had access to 6 or more visits (Table 1).

**Table 1:** Antenatal data regarding 3247 children residing in São Carlos, São Paulo, who were born in 2018

Gestational variable	*n* = 3247	%
**Access to antenatal care **^**1**^		
Did not have antenatal care	3	0.09
Not adequate	611	18.82
Adequate	2444	75.27
Not informed	189	5.82
**Number of antenatal visits**		
None	16	0.49
1–6	436	13.43
> 6	2754	84.82
Not informed	41	1.26

^1^Adequate antenatal care was that which began in the first trimester and had a minimum of six visits [27]

Source: [33]

Most children were born with Apgar score higher than six in the first minute (96.80%), by caesarean Sect. (57.56%) and weighing more than 2,500 g (91.62%). The analysis of type of birth according to socio-demographic aspects showed that caesarean section had a positive correlation and was more frequent in Caucasian women with stable marital relationships, over 34 years of age, and more than 11 years of education. Therefore, it was a more common occurrence in women of better social status. However, the type of birth was indifferent among women aged 20 to 34 years, with 8 to 11 years of schooling, and those who were not Caucasian or Brown. In adolescents, caesarean section was 78% less frequent than in the other age groups as a whole (OR = 0.22; CI = 0.17–0.29; *p* = 0.000); in women with less than eight years of education, it was 64% less frequent than in women with more than seven years of education (OR = 0.36; CI = 0.29–0.45; *p* = 0.000), and in Brown women, the frequency of caesarean section was 44% lower than in the other age groups as a whole (OR = 0.56; CI = 0.48–0.66; *p* = 0.000) (Table 2).

**Table 2:** Distribution of the type of birth according to sociodemographic aspects at São Carlos, São Paulo, 2018

**Sociodemographic variable**	**Type of birth**		**Statistic analysis** ^1^	**Total**
	**Caesarean section**	**Vaginal**		
**Marital status**				
Stable union	151 (59.07%)	105 (40.93%)	OR = 1.34; CI = 1.13–1.59; *p* = 0.001	2568 (79.09%)
No stable marital partnership	345 (51.49%)	325 (48.51%)		670 (20.63%)
Not informed	7 (77.78%)	2 (22.22%)	-	9 (0.28%)
**Age group (years)**				
< 20	78 (25.91%)	223 (74.09%)	OR = 0.22; CI = 0.17–0.29; *p* = 0.000	301 (9.27%)
20–34	1319 (58.13%)	950 (41.87%)	OR = 1.08; CI = 0.93–1.26; *p* = 0.316	2269 (69.88%)
> 34	472 (69.72%)	205 (30.28%)	OR = 1.93; CI = 1.61–2.32; *p* = 0.000	677 (20.85%)
**Education (years of education)**				
< 8	153 (36.00%)	272 (64.00%)	OR = 0.36; CI = 0.29–0.45; *p* = 0.000	425 (13.09%)
8–11	1075 (56.22%)	837 (43.78%)	OR = 0.87; CI = 0.76–1.01; *p* = 0.065	1912 (58.88%)
> 11	637 (70.46%)	267 (29.54%)	OR = 2.15; CI = 1.83–2.54; *p* = 0.000	904 (27.84%)
Not informed	4 (66.67%)	2 (33.33%)	-	6 (0.18%)
**Race/Ethnicity**				
Caucasian	1357 (61.37%)	854 (38.63%)	OR = 1.63; CI = 1.40–1.89; *p* = 0.000	2211 (68.09%)
Black	84 (60.87%)	54 (39.13%)	OR = 1.15; CI = 0.81–1.64; *p* = 0.422	138 (4.25%)
Asian	6 (66.67%)	3 (33.33%)	OR = 1.47; CI – 0.31–9.14; *p* = 0.741	9 (0.28%)
Brown	414 (47.21%)	463 (57.79%)	OR = 0.56; CI = 0.48–0.66; *p* = 0.000	877 (27.01%)
Native people	2 (33.33%)	4 (66.67%)	OR = 0.37; CI = 0.03–2.57; *p* = 0.411	6 (0.18%)
Not informed	6 (100%)	0 (0.00%)	-	6 (0.18%)
Total	1869 (57.56%)	1378 (42.44%)		3247

^1^Mantel-Haenszel two-tailed chi-square when number of occurrences >  = 5; Fisher's exact test when number of occurrences < 5

*OR* odds ratio, *CI* confidence interval

Source: [33]

When correlating the type of birth with antenatal attendance data, caesarean section was 19% more frequent in women with adequate antenatal care (OR = 1.19; CI = 1.01–1.40; *p* = 0.031), therefore, with a higher number of visits (OR = 1.47; CI = 0.08–87.09; *p* = 0.000). In this specific group, having had more than six antenatal visits contributed to increase by 47% the occurrence of caesarean section. Twin pregnancy was also a factor positively related to caesarean section (OR = 3.31; CI = 1.06–5.54; *p* = 0.000). The type of birth was indifferent to gestational age (Table 3).

**Table 3:** Distribution of the type of birth according to gestational aspects at São Carlos, São Paulo, 2018

Gestational variable	Type of birth		Statistical analysis ^2^	Total
	**Caesarean section**	**Vaginal**		
**Access to antenatal care** ^1^				
Did not have antenatal care ^2^	2 (66.67%)	1 (33.33%)	OR = 1.47; CI = 0.08–87.09; *p* = 1.000	3 (0.09%)
Not adequate	319 (52.21%)	292 (47.79%)	OR = 0.76; CI = 0.64–0.91; *p* = 0.003	611 (18.82%)
Adequate	1433 (58.63%)	1011 (41.37%)	OR = 1.19; CI = 1.01–1.40; *p* = 0.031	2444 (75.27%)
Not informed	115 (60.85%)	74 (39.15%)	-	189 (5.82%)
**Gestational age (weeks)**				
22–27	5 (45.45%)	6 (54.55%)	OR = 0.61; CI = 0.17–2.11; *p* = 0.416	11 (0.34%)
28–36	173 (60.92%)	111 (39.08%)	OR = 1.16; CI = 0.91–1.50; *p* = 0.231	284 (8.75%)
> 36	1670 (57.39%)	1240 (42.61%)	OR = 0.93; CI = 0.74–1.17; *p* = 0.559	2910 (89.62%)
Not informed	21 (50.00%)	21 (50.00%)	-	42 (1.29%)
**Number of antenatal visits**				
None	10 (62.50%)	6 (37.50%)	OR = 1.23; CI = 0.44–3.66; *p* = 0.689	16 (0.49%)
1–6	223 (51.15%)	213 (48.85%)	OR = 0.74; CI = 0.60–0.91; *p* = 0.003	436 (13.43%)
> 6	1625 (59.01%)	1129 (40.99%)	OR = 1.47; CI = 1.21–1.78; *p* = 0.000	2754 (84.82%)
Not informed	11 (26.83%)	30 (73.17%)	-	41 (1.26%)
**Type of pregnancy**				
Twin	87 (81.31%)	20 (18.69%)	OR = 3.31; CI = 1.06–5.54; *p* = 0.000	107 (3.30%)
Singleton	1781 (56.77%)	1356 (43.23%)		3137 (96.61%)
Not informed	1 (33.33%)	2 (66.67%)	-	3 (0.09%)
**Total**	1869 (57.56%)	1378 (42.44%)		3247

^1^Adequate antenatal care was that which began in the first trimester and had a minimum of six visits [27]

^2^Mantel-Haenszel two-tailed chi-square when number of occurrences >  = 5; Fisher's exact test when number of occurrences < 5; *OR* odds ratio, *CI* confidence interval

Source: [33]

With regarding to the birth conditions of the neonate an Apgar score lower than six in the first minute was 50% more frequent in vaginal births, with a statistical difference in relation to caesarean section (OR = 1.50; CI = 0.99–2.25; *p* = 0.050). On the other hand, being born by caesarean section was more frequent among babies with birth weight greater than or equal to 4,000 g (OR = 1.62; CI = 1.14–2.33 *p* = 0.007) (Table 4).

**Table 4:** Distribution of birth conditions according to type of birth in São Carlos, São Paulo, 2018

Weight at birth (grams)	Type of birth			Statistical analysis
	**Caesarean section**	**Vaginal**	**Total**	
< 1500	29 (61.70%)	18 (38.30%)	47 (1.45%)	OR = 1.19; CI = 0.66–2.19; *p* = 0.563
1500–2499	142 (63.11%)	83 (36.89%)	225 (6.93%)	OR = 1.28; CI = 1.97–1.70; *p* = 0.081
2500–3999	1599 (56.50%)	1231 (43.50%)	2830 (87.86%)	OR = 0.71; CI = 0.57–0.87; *p* = 0.001
> = 4000	99 (68.28%)	46 (31.72%)	145 (4.47%)	OR = 1.62; CI = 1.14–2.33; *p* = 0.007
**Apgar in the first minute**	**Type of birth**			**Statistical analysis**
	**Caesarean section**	**Vaginal**	**Total**	
< 6	46 (47.92%)	50 (52,08%)	96 (2.96%)	OR = 1.50; CI = 0.99–2.25; *p* = 0.050
6–10	1821 (57,64%)	1322 (42.36%)	3143 (96.80%)	
Not informed	2 (25.00%)	6 (75.00%)	8 (0.25%)	-
Total	1869 (57.56%)	1378 (42.44%)	3247	

*OR* odds ratio, *CI* confidence interval

Source: [33]

Other antenatal care quality indicators are the maternal and infant mortality rates. It was observed that the Perinatal Mortality Rate had the highest coefficient among the indicators related to infant mortality (Table 5).

**Table 5:** Indicators related to maternal and child mortality in São Carlos, São Paulo, 2018

**Indicator**	**Value (**per thousand live births)
Foetal mortality rate	7.0
Early neonatal mortality rate	6.8
Perinatal mortality rate	13.8
Late neonatal mortality rate	1.8
Post-neonatal mortality rate	1.5
Infant mortality rate	10.2
^*^ Maternal mortality rate	61.6

^*^Note: Maternal mortality rate is per 100,000 live births instead per thousand live births

The qualitative study pertaining to the perspectives and experiences of women identified three thematic categories: "Brief, child-centred clinical assessment", "(Dis)satisfaction in interactions with professionals" and "Information barriers". The sociodemographic characteristics of these women is shown in Table 6.

**Table 6:** Distribution of pregnant women interviewed according to sociodemographic aspects

Sociodemographic data	N	%
**Age group (Years old)**		
18–25	20	67
26–30	7	23
31–35	3	10
**Number of previous pregnancies**		
At least 1	17	57
None	13	43
**Planned pregnancy**		
Yes	9	30
No	21	70
**Marital status**		
Single	14	47
Married	12	40
Uninformed	2	6
**Working condition**		
Paid	18	60
Unemployed	10	33
Uninformed	2	7
**Level of education**		
High School	19	63
Elementary School	5	17
Uninformed	6	20
**Race/ethnicity**		
Caucasian	25	83
Non-Caucasian	5	7

In the first category of the analysis of the pregnant women’s interviews, "Brief, child-centred clinical assessment", the meaning of the ANC is synonymous of a quick medical appointment. The nurse is only responsible for some technical procedures, such as blood pressure and body weight monitoring. The following excerpt shows what the ANC visits represent for these pregnant women:

Oh, I like to come and listen to her (child’s) little heart, to see if everything is ok, so that’s the cool thing, there’s nothing else (laughs) (G14).

In the category "(Dis)satisfaction in interactions with professionals", there are professional behaviours interpreted as disinterest and disrespect, with emphasis on the long waits to receive care, and the short duration and little interaction during the visits. In these situations, ANC is qualified as superficial, the motivation for visits is scarce and almost sustained by the obligation to come, as the following speech reinforces:

I think it is a lack of responsibility on her part (doctor) because it is scheduled for the right time there, it always takes a while, we have other things to do and we have to stop doing them to be able to wait for her to come and see the patients. Then, when she sees us, it takes only seconds (G05).

The category “Information barriers” brings the expectation of women to receive information about their needs, especially relating to birth and tubal ligation. There is no space to address their fears of a vaginal birth, which are perpetuated in the conversations between them in the waiting rooms of antenatal visits. When they want a tubal ligation, the participants are led to opt for a caesarean section, a private payment is made (a type of financial agreement with the doctor) so the tubal ligation is made possible and they do not receive any information on other possibilities to request the tubal ligation, as expressed in the following excerpts:

I just talked to him (doctor), I'm going to have a caesarean because I heard so much about how we suffer a lot, there are so many risks, there are people who have recently had a baby in the family and they (doctors) hold until the last moment to perform the caesarean, even heard of a baby who has died, so you end up being afraid of waiting until the last day of pregnancy (G02).

She (doctor) explained that paying privately, I can choose the caesarean section to get the tubal ligation after the baby is born [...] (G04).

The lack of empathy at ANC visits is noticeable and discourages women to voice their doubts and/or needs resulting in their withdraw from asking questions or interacting. Added to this is a past of difficult relationships with the professional and/or service that limits the exposure of their informational needs, as expressed by the pregnant woman:

[...] sometimes she (doctor) asks me if I am afraid of anything and then when I don't understand something and ask a question, she gets like this (grimace, long pause) and starts to explain. It seems like she would say "you are dumb, huh". I didn't ask any more questions (G06).

The qualitative study pertaining to the perspectives and experiences of the health professionals identified two thematic categories: "Understandings of antenatal care" and "Limitations of nurses in the work process and in antenatal care”.

When looking at what was revealed in the voice of eight female nurses, the first category "Understandings of antenatal care", pointed out the importance of antenatal care and of nursing for a comprehensive and good-quality care:

[…] when nurses act [...] doing all that nursing consultation, when you have the capacity to be in all consultations [...] it is very important to approach all aspects, the breasts, vaginal question, how she is, question about signs and symptoms, all family question [...] depending on how nurses act, it can avoid several complex things, both for the mother and the baby.

The thematic category "Limitations of nurses in the work process and in antenatal care" addresses the fact that even with the legal permission for promoting and carrying out antenatal care, nurses avoid taking this on as much as possible, out of fear, insecurity, and because they believe they do not have the training needed. This seems to be the perspective of several nurses as exemplified by the following speech:

[...] the first thing I see is that most nurses do not feel able to deliver antenatal care, they think that this is not their job, so many do not feel capable, there is also a lack of training, many end up working in other areas, [...] there is a lack of knowledge [...] I notice a lot of resistance from nurses, at least in this city[...]. The nurses at the family health unit yes, those at the BHU no.

The lack of protocols, training, and time to provide care, on top of management duties, make these professionals not take on ANC in an active way as shown in the following excerpt:

Here, in the BHU, I don't have conditions to conduct the antenatal care intercalated with the doctor's [...] we don't only have this activity, so, what we can do are these two services (rapid tests and first consultation).

The nurses recognized primary healthcare as a gateway to the public health system and a strategy for health promotion within the scope of ANC. They perceived the ANC as a potential space for their professional development, however, they identified obstacles to their autonomous work in this area. Nurses’ limitations to work with antenatal care are due to a lack of technical knowledge in midwifery. In addition to the dedication of a lot of their time to the administrative aspects of the health unit, which reduced the possibility of them being closer to women, which in turn reduced the chance of meeting women’s needs, especially regarding information about birth.

## Discussion

This work allowed an analysis of ANC in a Brazilian city located in the state of São Paulo (Brazil). The results raised relevant issues regarding the loss of opportunity of the ANC in contributing to the reduction of caesarean sections. A statistically significant correlation was found with increased antenatal visits and caesarean section which is directly opposed to the objectives of improving maternal and child healthcare in Brazil.

Antenatal care, as found in this study, was reduced to procedures, exams and ultrasonography aimed at the foetal health. The meaning and relevance of ANC care are incomplete, especially due to the fragilities in the relationship with the professionals since it happens over quick visits and is limited to procedures. Pregnant women have little opportunity for dialogic interactions, with the undervaluation of their needs and complaints. There is a prevalence of experiences perceived as contempt, hierarchical, anti-dialogical and disrespectful relationships. Women are not heard and their autonomy is disregarded, especially on issues surround birth.

For nearly 30 years, most countries affiliated with the World Health Organization have participated in the Risk-Free Maternity Initiative (RFMI), aiming to raise awareness and promote action at global and country levels to achieve safer pregnancies and births for women and their newborns [34] and a positive pregnancy experience, which implies prioritising woman-centred healthcare, the well-being of women and families, and positive perinatal and maternal outcomes [3].

Humanization in obstetric care has multiple meanings and indicates a change in care practices during pregnancy, birth, and the postpartum period [1, 30]. The Stork Network was constituted as a program of the Brazilian Ministry of Health with the main objective of promoting changes in the maternity care model in the country, expanding access and improving quality of pregnancy, birth, postpartum and reproductive planning services in order to ensure the reduction of maternal and child mortality throughout the country.

The concept of humanization in the Brazilian maternity care context has origins in the women's movement in the 80's and 90's. The main demand was for a transformation of the biomedical model, for respectful care, based on autonomy, protagonism and rights. Thus, the concept of humanization starts to be incorporated into national policies for maternity care, and in this scenario humanization is directly related to care practices, access and quality of health services [1].

The high rates of caesarean section in the private sector and among Caucasian women show the inequalities of class and race/ethnicity in the country. It is important to say that the desire for a caesarean section in Brazil is also linked to a way of escaping a vaginal birth full of unnecessary interventions and violence, such as the practice of episiotomy, the Kristeller manoeuvre and the non-compliance with the law that assures a birth companion to women. The women's movement in Brazil has demonstrated the impact of obstetric violence on the experience of childbirth and on women’s lives. Organizations such as Artemis^1^ have acted on different fronts and are committed to eradicating all forms of violence against women. The debate about obstetric violence has been gaining ground in Brazil, as exemplified by the production of the video documentary Violência Obstétrica: a voz das brasileiras^2^ (Obstetric Violence: the Brazilian’s voices). All efforts of the government and women's movements throughout the country have sought to transform this scenario through the discussion of birth and the concept of humanization of birth comprehensively [35].

Therefore, humanization in pregnancy and childbirth should not be reduced to something contrary to a biomedical and patriarchal model of care, but should be seen as an essential approach to care, which must be adopted by all health professionals who care for women during pregnancy and birth. Through concept analysis the authors identified three attributes related to humanization in the context of pregnancy and birth: human interaction, benevolence and protagonism. The authors emphasize the importance of not conceptualizing humanized care as low-risk care. Humanization can be understood as a care structure that integrates a broad view of the dimensions of women's lives, accompanied by the implementation of practices and interventions, when necessary. In this sense, humanization includes care based on the best scientific evidence and that, at the same time, meets the individual and specific needs of women [36].

In Brazil, caesarean sections show tendencies of increasing, as in 2000 it represented 38% of all births, and in 2019 it was 56% [37]. The medicalisation of birth is a reality and antenatal care demonstrates limited impact in transforming it. Vaginal birth care has not incorporated scientific evidence, obstetric violence still occurs and is present in birth scenarios [9]. The current model of antenatal care does not seem to be empowering women to make well-informed decisions.

The development of a bond with the health professionals is essential to promote access to the health service, and for the women to feel welcomed and secure. These statements are discussed in the national documents and guidelines for antenatal practices [9]. Barriers in access, low quality of antenatal and birth care do occur and were seen in this study. So were adverse outcomes for women and newborns and little change in infant and maternal morbidity and mortality rates [38].

It is known that problems in antenatal care influence perinatal mortality more than post-neonatal mortality, while social problems significantly influence post-neonatal mortality. A pattern of infant mortality concentrated on the perinatal mortality rate (when the value of this indicator is higher than the others related to the death of children under one year old) indicates the need to invest more in the assistance model than in social conditions. The results suggest that there is still a lack of actions to improve the model of care at the studied place and that these actions are more necessary now than investments in social improvements, at least in relation to maternal and child health.

This need for investment in the model of care is connected to maternal and child health indicators. Maternal mortality in Brazil has been slowly decreasing, while perinatal mortality has increased, reinforcing the responsibility of administrators in reducing maternal and infant mortality through actions aimed at reducing health inequities [39–41].

On the other hand, a study done in a city in the Northeast region of Brazil [42] indicated that ANC had initiation of antenatal care; insufficient number of visits; little guidance, including on the lack of guarantee of a room in the maternity hospital at the time of birth which can lead to women having to go from one hospital to another in search of an available hospital bed at the time of birth; lack of prioritisation of higher risk pregnant women and problems in the articulation with other maternal and child health services. What we wish to highlight is that, without considering the quality indicators of the ANC and by devaluing the needs of pregnant women, not placing them in the centre of the care, we cannot provide a positive pregnancy experience and prevent maternal and neonatal morbidity and mortality.

Comprehensive healthcare, the guiding axis of this discussion, a principle taken by the government to guide healthcare in Brazil, presupposes a healthcare and a sectorial management that recognise the autonomy and the cultural and social diversity of people and populations [43]. When considering the other principles assumed in healthcare, universality urges us to build access for all: the principle of equity demands that we agree with everyone on what each person needs, and the principle of comprehensive care urges us to know and to do the "what" and "how" to respond universally to the needs of each person [26–29].

In this sense, comprehensive healthcare, as one of the principles that constitute the UHS, operates in two fields: the field of law, the guarantee of access at all levels of care, health promotion and disease prevention. The other field concerns the integral understanding of people, considering the bio-psychosocial aspects, in order to overcome the fragmented view and reduced to the biological aspect. In Brazil, comprehensive care is seen as a common thread the organisation of health services so that the different knowledge of health professionals, users and the community is in constant exchange and, thus, can compose the management plans, which directly impacts on the health practices and the production of care [44].

According to the findings of this study, antenatal care shows a fragmented care permeated by challenges that involve the need for change in management, in the performance and in the ongoing training of professionals, as well as in the guarantee of rights of the women. In this sense, the quality of antenatal care needs to be based on comprehensive care. The National Survey of Birth in Brazil [45] showed that, from the quantitative point of view, antenatal care in the country is adequate, with guaranteed wide coverage and adequate number of visits. However, the data analysed in the survey shows that from a qualitative point of view, ANC in Brazil is weak. There is fragmentation of the network and the lack of implementation of health education actions.

This study used the secondary research database of the SUS General Ombudsman's Office, with information on 253.647 women, and showed the vulnerabilities of black women, who appear in worse conditions in terms of socioeconomic characteristics and have a higher number of teenage pregnancies among other aspects that affected the way these women were cared for during pregnancy, birth and the postpartum period [46].

One of the main obstacles to a comprehensive antenatal care is the performance of the healthcare team. For this, it is necessary to have a team with an interprofessional approach, with the valorisation of the performance of professionals from different backgrounds and competencies in health. According to the data of the studied context, antenatal care remains mostly centralised in the medical team, with little space and autonomy for the work of other health professionals. In contrast to the ANC in the British healthcare system [47], which values the role of midwives, the Brazilian primary healthcare needs to insert and expand the role of these professionals in maternity care. According to these authors, this action is powerful in guaranteeing women's rights, reducing obstetric violence and the number of caesarean sections. This argument corroborates the results of the Cochrane review [48] on midwife/nurse-led models of care. The review found that women who received midwife-led care were less likely to experience intervention and were more satisfied with the care they received. The review also showed a reduction in adverse outcomes in this model of care when compared to traditional models.

The perception of care in the relationship between professionals and women was directly connect with the evaluation of the ANC. All acts are symbolically interpreted as translators of interest (or not) of the professional and lead to a positive or negative evaluation. In this sense, the perceptions revealed in this study denote little relevance and meaning of ANC, with relational insufficiencies, marked by being identified by pregnant women’s speeches of minimal dialogue and hierarchical in nature. Pregnant women also felt their needs were disregarded.

The participants of this study did not have a distinctly positive experience of care during prenatal visits. The appointments showed little possibility to share doubts and were limited to physical examinations. The symbolism of ANC care emerges as compliance with protocol measures, not valuing pregnant women, and perhaps family interactions. In this sense, the ANC needs to be valued as an encounter that requires an individualised look at the woman, genuine dialogue, intersubjective exchanges, to rescue the meaning of care itself [26, 27, 49]. Therefore, it implies welcoming the inter-subjectivity in each encounter, with efforts to apprehend and attend to the singularity manifested there [19].

The results also allowed us to conclude that there is an insufficiency of epidemiological data and that the few that are available, are not used by the city as indicators to guide the planning of healthcare actions. Therefore, there are flaws in the municipal management of information systems, which makes it impossible to adequately manage antenatal healthcare in this city, negatively impacting the quality of care.

This study points to three structuring factors for higher quality ANC: broadening the scope of the clinic, investing in egalitarian relationships, and ensuring unique informational support. They are all intertwined and have, in the attitudinal of the professional, a key determinant. The focus on egalitarian dialogue and qualified listening requires investments in professional and continuing education, and the critical and reflective exposure of clinical narratives can be a resource, as well as the need for management to understand the potential of nursing in ANC. It is also necessary to recognise the importance of healthcare networks and the model of lines of care as strategies to overcome the fragmentation of care and management, in the search for the challenges and achievements of comprehensive care for pregnant women and their families, which will require reviewing work processes, relationships and interactions and a support and commitment from the municipal management to the critical nodes identified.

Understanding the factors that affect the quality of antenatal care requires the use of diversified tools, given the complexity of the phenomenon. The different methods and the critical reflective analysis carried out allowed an understanding of the quality of care articulating its technical and inter-subjective dimensions. Thus, antenatal care is expressed in a daily routine that is not always welcoming to pregnant women and is often marked by power relations. This analysis highlighted the limits of the biomedical paradigm and the importance of an expanded conception of care to make the subjects and inter-subjective processes the centre of attention in the production of care and in the way of doing management.

The study showed that the ANC has weaknesses in the interactions between pregnant women and professionals, with the prevalence of the biomedical model, focused on complaint-conduct, with dissatisfaction and little participation of nurses from Basic Health Units in the care provided, which had repercussions on antenatal care quality, with reduced chances of making it effective as a welcoming space focused on the needs of pregnant women. It is concluded that the ANC has not favoured the decrease in the incidence of caesarean sections, which remains high, and birth care indicates the need for further investigation.

The contributions of the study permeate the appreciation of the quality of inter-subjective interactions in the care of pregnant women and the construction of teamwork. The achievements and challenges to the comprehensive healthcare reveal the need to prioritise a care focused on the needs of the pregnant woman and her family and that, thus valued, can be the guide for the reorganisation of the work process in health aiming at changes in professional attitude, articulation of healthcare networks, cooperation between health and social sectors, participation, and social mobilisation.

The limitations of these study, the lack of good epidemiological records is pointed out, compromising not only the study, since a situational analysis could be made with data from DATASUS; however, this "blackout" of data also compromises actions and public policies for the city itself. The reports of women and professionals inserted in Basic Health Units, therefore, only in a single care model, were another limitation of this study. However, the precepts of ANC should be manifested in all care spaces, regardless of the care model. In addition, the discussions outlined here showed the potential to explore, in depth, meanings and senses, despite not aiming at generalisations.

Finally, the answers to the "how" and "why" of the case studied (quality of the ANC) allowed us to affirm the need to re-signify health and attitudinal practices in the direction of dignified, respectful reception, aligned with the right to health, regardless of their culture, financial and psychological conditions. Recognising not only the health, but the life needs of these women, in an encounter that can take place in the different spaces of antenatal care, is urgent. The ANC really needs to become an opportunity for healthcare providers to engage with women and their families and provide a positive pregnancy experience.

## Data Availability

The quantitative datasets used and/or analysed during the current study are available according to material and methods section'. The full transcribed interviews, code and meta data about qualitative studies are not publicly available due to the qualitative nature but are available from the corresponding author upon request.

## Abbreviations

ANC: Antenatal Care
BHU: Basic Health Units
CI: Confidence interval
DATASUS: Department of the Unified Health System
FHU: Family Health Units
OR: Odds ratio
SINASC: Live Births Information System
UHS: Unified Health System
FAPESP: São Paulo Research Foundation

## Footnotes

1 https://www.artemis.org.br/historia

2 Popular video documentary produced by Bianca Zorzam, Ligia Moreiras Sena, Ana Carolina Franzon, Kalu Brum, Armando Rapchan. https://www.youtube.com/watch?v=RMfAvgfqd9g
